# Feasibility and Enjoyment of Exercise Video Games in Older Adults

**DOI:** 10.1093/geroni/igaa057.1838

**Published:** 2020-12-16

**Authors:** Sara Freed, Briana Sprague, Lesley Ross

**Affiliations:** 1 Penn State University, University Park, Pennsylvania, United States; 2 University of Pittsburgh, Pittsburgh, Pennsylvania, United States; 3 The Pennsylvania State University, University Park, Pennsylvania, United States

## Abstract

Interventions using exercise video games, or exergames, have shown short-term cognitive and physical benefits to older adults, though long-term effects are less promising. Enjoyment of exergames may promote exergame use after the intervention period, though little work has examined older adults’ views of exergames before and after gameplay experience. We invited 20 older adults between 65 and 84 years of age (M=73.30, SD=5.95) to play two Xbox Kinect games, Just Dance and Kinect Sports Rivals, for twenty minutes. In our presentation, we will present qualitative and quantitative findings of this pilot study, including findings that older adults reported that they were not likely to play similar exergames in the future and that they did not find the exergames to be more fun compared to other ways of exercising. We will discuss implications for game design and research relevant to game developers, manufacturers, and researchers. Part of a symposium sponsored by Technology and Aging Interest Group.

